# Association of ability to rank sweet and fat taste intensities with sweet and fat food propensity ratios of children, adolescents and adults: the I.Family study

**DOI:** 10.1007/s00394-024-03538-0

**Published:** 2024-12-11

**Authors:** Hannah Jilani, Timm Intemann, Gabriele Eiben, Fabio Lauria, Lauren Lissner, Nathalie Michels, Dénes Molnár, Luis A. Moreno, Valeria Pala, Michael Tornaritis, Toomas Veidebaum, Wolfgang Ahrens, Antje Hebestreit, the I.Family consortium

**Affiliations:** 1 Institute for Public Health and Nursing Research–IPP, University of Bremen, Bremen, Germany; 2 Leibniz Institute for Prevention Research and Epidemiology - BIPS, Achterstr. 30, 28359 Bremen, Germany; 3 Department of Public Health, School of Health Sciences, University of Skövde, Skövde, Sweden; 4 Institute of Food Sciences, National Research Council, Avellino, Italy; 5 Department of Public Health and Community Medicine, University of Gothenburg, Sweden Gothenburg,; 6 Department of Public Health, Ghent University, Ghent, Belgium; 7 Department of Pediatrics, Medical School, University of Pécs, Pécs, Hungary; 8 GENUD (Growth, Exercise, Nutrition and Development) Research Group, Faculty of Health Sciences, University of Zaragoza, Instituto Agroalimentario de Aragón (IA2), Instituto de Investigación Sanitaria de Aragón (IIS Aragón) and Centro de Investigación Biomédica en Red Fisiopatología de la Obesidad y Nutrición (CIBEROBN), Zaragoza, Spain; 9 Department of Preventive and Predictive Medicine, Fondazione IRCCS Istituto Nazionale dei Tumori, Milan, Italy; 10 Research and Education Institute of Child Health, Strovolos, Cyprus; 11 Department of Chronic Diseases, National Institute for Health Development, Tallinn, Estonia

**Keywords:** Food consumption, Sweet and fatty taste, Taste intensity

## Abstract

**Purpose:**

It is assumed that sensory taste perception shapes food choices and impacts dietary intake. However, this has rarely been studied in free living subjects of different age-groups with standardised methods. The present study investigated the association of the ability to rank sweet and fat taste intensities with consumption frequency of sweet and fatty foods in children, adolescents and adults from eight European countries.

**Methods:**

In total, 461 children, 421 adolescents and 612 adults from the IDEFICS/I.Family cohort participated in sensory sweet and fat intensity rating tests. Sweet and fatty food consumption frequencies were assessed using a food frequency questionnaire. The association between the ability to rank sweet and fat intensity with sweet and fatty food consumption frequencies was estimated using linear mixed regression models adjusting for weight status, country, sex, age and family affiliation.

**Results:**

Across all age groups, the largest proportion of participants had medium sweet and fat taste intensity ranking abilities. The next largest proportion had low sweet and fat taste intensity rating abilities, while the smallest proportion had high intensity rating abilities to sweet and fat taste. A negative association of sweet and fat taste intensity ranking ability with sweet and fatty food consumption frequencies was found for children. In adolescents, the association was positive. In adults, there was no association.

**Conclusion:**

It seems that the association of taste intensity ratings with food consumption frequencies during adolescence differs from the associations in children and adults. This could be due to hormonal changes during puberty, growth and maturation. Thus, further research focussing on maturation processes in association with taste perception during adolescence may be required.

**Supplementary information:**

The online version contains supplementary material available at 10.1007/s00394-024-03538-0.

## Background

Overweight and obesity are a major public health problem in Europe [1]; unhealthy dietary patterns are an important risk factor characterized by high intakes of sugary and fatty foods [2] contributing to high energy intakes [3]. Therefore, dietary guidelines throughout Europe recommend to limit the consumption of such foods and drinks [4]. Nevertheless, the consumption of energy dense foods and drinks remains high (e.g [5]). The determinants for a high consumption frequency can be various. Especially in children, taste preference is an established factor exerting impact on food preference and food consumption [6, 7]. Taste perception can not only be described by preferences.

Taste perception can also be characterised by *taste sensitivity* and *perceived taste intensity*. Taste sensitivity is assessed by measuring detection or recognition thresholds using aqueous solutions (e.g [8]). Nevertheless, concentrations of tastants in real foods are higher than the concentrations to assess taste sensitivity. Therefore, it can be argued that detection or recognition thresholds might have no or a smaller impact on actual food consumption. Still, former studies focussing on taste sensitivity found associations with food consumption. For example, in adults with and without overweight/obesity a low-fat diet given during an intervention increased fat sensitivity [9, 10] and accordingly in individuals with lower fat sensitivity consumption of more fatty foods was observed [11, 12]. In children and adults who were more sensitive to bitter taste, Negri et al. observed avoidant consumption of bitter tasting vegetables, with a stronger effect for children than for adults [13]. Further, children with high fat sensitivity showed lower preferences for fatty foods [14]. Another study in young adults, observed a positive correlation between taste sensitivity and perceived taste intensity of suprathreshold concentrations of sweet, salty, sour, umami and fatty [15]. Perceived taste intensity of suprathreshold concentrations refers to the individual perception of taste intensity of concentrations above recognition, i.e. those concentrations that occur in real foods. The perceived taste intensity differs between individuals and might also be associated with food consumption. In an intervention study fostering a low-sugar diet in healthy adult men and women, the perceived sweetness intensity increased in the intervention group compared to the control group [16].

Due to the maturation status of the taste apparatus, taste perception differs between children, adolescents and adults. Most studies suggest that children are less taste sensitive compared to adults, at least when taste sensitivity is measured using thresholds [17, 18], and that adolescents are more taste sensitive than children [18, 19]. Less is known about the differences in perceived taste intensity between children, adolescents and adults. One study by Mennella et al. (2012) described that mothers and children were equally able to rate the creaminess intensity of specific fat concentration, but mothers could discriminate sweet taste intensity better than children [20].

In I.Family study, we measured the ability of children, adolescents and adults to discriminate the intensity of creaminess (fat content) and sweetness (sugar content) in standardised pudding samples as a food matrix. Real food as test matrix were used to increase acceptability, as this reflects children’s real-world conditions. Further, we used a semi-liquid food matrix, as it facilitates diffusing the taste in the oral cavity better than solid foods. Consequently, a semi-fluid matrix possibly allows the taste to remain longer in the oral cavity compared to completely liquid foods/drinks. The present study uses the term “perceived fat intensity” for the multisensory lipid perception experienced when tasting fat, including mouthfeel, creaminess and taste. In addition, we assessed the tendency to choose foods high in sugar or fat (sweet and fat propensity ratios) from self-reported data of a qualitative food frequency questionnaire.

The ultimate aim of this epidemiological study was to investigate the association of the ability to discriminate sweetness and creaminess with sweet and fat propensities ratios separately for the different age groups across Europe.

## Methods

### Study design and participants

The I.Family study, a pan-European cohort study in eight European countries (Belgium, Cyprus, Estonia, Germany, Hungary, Italy, Spain and Sweden) [21], was conducted between October 2014 and May 2016. A subsample of children, adolescents and their parents (biological and non-biological mothers, fathers and miscellaneous guardians) were invited to the examination of sensory taste perception. In total, 951 children and adolescents and 679 parents participated in an examination including tests on sensory taste perception and assessment of dietary intake. Subjects with missing data in any of the covariates or the response variables were excluded from the analysis. Each study centre obtained ethical approval from the local institutional review board. Besides the oral information given, all participants above 12 years gave a written consent and parents gave a written consent on behalf of their children below 12 years in addition to the oral consent of their children.

### Questionnaires

Participants’ age, sex and country of residence were assessed via questionnaires. The parents provided information on their level of education. For each parent the highest educational level according to the International Standard Classification of Education (ISCED) ranging from 0 (low education) to 8 (high education) was recorded [22]. For the present analysis, the educational level was grouped into three categories; ‘low education’ (ISCED level 0–2), ‘medium education’ (ISCED level 3–5) and ‘high education’ (ISCED level 6–8). The highest education level of a household (e.g. the highest education level achieved by the parents) was used as a proxy indicator for socio-economic status of the family. Further, for this analysis it was assessed if participants were living in the same household via a family ID to account in the analysis for similarities among household members (see statistical analysis).

### Dietary assessment

An adapted version of the Children’s Eating Habit Questionnaire containing a Food Frequency Questionnaire (FFQ) used in the IDEFICS study was completed by the participants. This is a validated [23] instrument which provides reproducible results [24], completed by parents for children below an age of 12 years as they are unreliable reporters of their diet [25]. The FFQ version used in the I.Family study was identical to the IDEFICS study for children, but the food groups alcoholic beverages and coffee were added for adolescents and adults. The FFQ used in this analysis contained 59 items including fatty foods (fried potatoes, whole fat milk, whole fat yoghurt, fried fish, cold cuts/sausages, fried meat, fried poultry, fried eggs, mayonnaise and mayonnaise based products, cheese, chocolate- or nut-based spread, butter/margarine on bread, oil, nuts and seeds, salty snacks, savoury pastries, chocolate-based candies, cake/pudding/cookies and ice cream) and sweet foods (fresh fruit with added sugar, fruit juices, carbonated sugar sweetened drinks, sugar sweetened drinks not carbonated, sweetened coffee, sweetened tea, sweetened or sugar added breakfast cereals, sweetened and/or flavoured milk, sweetened and/or flavoured yoghurt, sweet spreadable (jam, honey, chocolate or nut based), and snacks (chocolate-based candies, non-fat candies, cake/pudding/cookies, ice cream). The response categories were ‘never/less than once a week’, ‘1–3 times a week’, ‘4–6 times a week’, ‘1 time/day’, ‘2 times a day’, ‘3 times a day’ and ‘I have no idea’. The weekly consumption frequencies of the aforementioned fatty foods, aforementioned sweet foods and all foods included in the FFQ were summed up. Sweet and fatty food consumption frequencies were calculated based on the relative frequency of consumption of the sweet or fatty food compared to the frequency of consumption of all foods included in the FFQ [26]. This calculation resulted in two continues indices; one for fatty foods and one for sweet foods. These two propensity ratios reflect proportions of fatty or sweet foods in the whole diet (Laurens paper). In this calculation individuals for whom more than 50% of the answers were missing or not known were excluded.

By using sweet and fat propensity ratios the sweet and fatty food consumption frequency is standardised on frequency of all consumed foods. The relative validity for this approach was described by Lissner et al. [27].

All instruments and recording sheets were developed in English, translated to the national language including national examples and then back-translated to check for translation errors.

### Anthropometry

Weight status (underweight, normal weight, overweight and obese) of the children was calculated according to Cole and Lobstein [28]. First, the children’s BMI was calculated and converted to age- and sex-specific z-scores. Using age- and sex-specific cut-offs published by Cole and Lobstein. children were then classified into underweight/normal weight and overweight/obese (weight status). The cut-offs for overweight were for boys the 90.5th and for girls the 89.3rd percentile curve [28].

Weight status for adults was categorized as follows: Adults with a body mass index (BMI) value < 18.5 kg/m2 were classified as underweight, adults with BMI between 18.5 kg/m^2^ and 25 kg/m^2^ were classified as normal weight, adults with BMI between 25 kg/m^2^ and 30 kg/m^2^ were classified as people with overweight and finally adults with BMI > 30 kg/m^2^ were classified as people with obesity [29].

### Assessment of sweet and fat intensity ratings

All participants included conducted a sweet and fat taste intensity test. Cold whipped vanilla flavoured pudding (RUF™ Schlemmer Crème, Vanille Geschmack) was chosen to assess perceived sweet and fat taste intensity [20]. For sweetness, three standardised pudding samples with concentrations of 14.5 g, 24.1 g and 36.2 g sugar per 100 g were prepared. For fat, three standardised pudding samples were prepared by varying the proportion of skimmed milk and cream containing 3.1%, 6.8% and 14.1% wt/wt fat. All pudding samples were presented at room temperature. Participants restrained from eating for at least 1 h before the taste intensity test. The pudding samples were presented under red light in order to mask colour differences. To assure a standardised test procedure across all 8 countries a central training was conducted. All test materials (e.g. all pudding samples, cups, cardboards and scales) were prepared centrally and then shipped to every centre.

A cardboard with a game-like illustration [30] was used to assess perceived taste intensity and taste preference ratings. For all participants, a 9-point-scale that was printed on the board was used to assess sweet and fat intensity ratings. Before participants started with the actual taste intensity test, the scale was explained in easy language and its handling was practised with the participant: using the 9-point-scale, three cards with different colour intensities (ranging from dark grey to light grey) had to be ranked for colour intensity on the scale [31]. Then, the participants rinsed their mouth with distilled water. Participants were also asked to rinse their mouth between the two taste modalities. Further, they were invited to rinse whenever they needed to. The test started either with sweet or fat in a random order. The three samples were presented in different and counterbalanced order in 20 ml plastic cups. After the participants tasted one sample of the puddings they rated the sweetness or creaminess intensity on the 9-point-scale. Intensity ratings obtained from the 9-point scale ranged from 1 (low intensity) to 9 (high intensity).

### Intensity ratings

According to the approach by Mennella et al. [20], the ability of each participant to rank the three pudding samples in the right order was used to determine his/her ability to discriminate foods that differ in sweetness or creaminess with a score of 0, 1 or 2. The value of 2 was assigned if all pudding samples were ranked correctly from lowest to highest concentration. The value of 1 was assigned if 1 pudding was ranked correctly and the other 2 incorrectly. And 0 was assigned if no sample was ranked correctly.

### Statistical analysis

The characteristics of the study sample were presented using descriptive measures for children, adolescents, adults and the complete study sample separately. Children were defined as 6.0 to 12.0 years, adolescents as older than 12 to 18 years and adults as older than 18 years. The distributions (N and %) of the discrete variables and means, standard deviations (SD) and the upper and lower quartiles of continuous variables were calculated.

For the analyses of associations, the exposure variables (sweet sensitivity and fat sensitivity) were dichotomised as follows: the lowest category of the exposure variables was chosen as reference category (dummy coded as 0) and the medium and high categories were combined (dummy coded as 1). To investigate the association between the ability to discriminate sweetness (exposure) and sweet propensity ratio (outcome) as well as between the ability to discriminate creaminess (exposure) and fat propensity ratio (outcome), linear mixed regression models were used. The analyses of associations were conducted separately for children, adolescents and adults. Family affiliation was used as random effect in linear mixed regression models to account for similarities between family members. As all analyses were conducted separately for children, adolescents and adults, therefore, were no child-parent-pairs in the analyses. Family affiliation accounted for similarities between siblings and for spouses due to assortative mating. Further, the linear mixed regression models included age, sex, country, weight status, and level of education (ISCED) as covariates. For adolescents and adults, the analysis was also adjusted for smoking status. Effect estimates of sweet and fat intensity ranking for sweet and fat propensity ratios, which can be interpreted as estimated adjusted mean differences between intensity ranking groups, and corresponding 95% confidence intervals (CI) were calculated. All analyses were conducted using SAS software version 9.3.

## Results

### Study sample

The study sample consisted of 461 children (mean age 10.6 years, ranging from 7.9 to 12.0 years), 421 adolescents (mean age 14.1 years, ranging from 12.3 to 17.3 years) and 612 adults (mean age 44.0 years, ranging from 29.7 to 63.4 years). The sex ratio among children and adolescents was almost balanced whereas among adults more than 73% were female. Approximately ¼ of the children and adolescents were overweight or obese. In adults, more than 50% were overweight or obese (Table 1).

**Table 1:** Characteristics of the study sample (total number (N) and percentages (%) or mean, standard deviation (SD) and first and third quartiles (Q1; Q3)) given by age groups

	Children	Adolescents	Adults	Total
	N (%)	N (%)	N (%)	N (%)
	461 (30.9)	421 (28.2)	612 (41.0)	1494 (100.0)
Female	230 (50.1)	234 (55.6)	467 (76.3)	931 (62.3)
Country				
Belgium	64 (13.8)	52 (12.4)	52 (8.5)	168 (11.2)
Cyprus	52 (11.3)	42 (32.8)	34 (5.6)	128 (8.6)
Estonia	81 (17.6)	63 (15.0)	126 (20.6)	270 (18.1)
Germany	34 (7.4)	54 (12.8)	72 (11.8)	160 (10.7)
Hungary	57 (12.4)	80 (19.0)	108 (17.7)	245 (16.4)
Italy	59 (12.8)	41 (9.7)	73 (11.9)	173 (11.6)
Spain	48 (10.4)	28 (6.7)	55 (9.0)	131 (8.8)
Sweden	66 (14.3)	61 (14.5)	92 (15.0)	219 (14.7)
Overweight/obesity^a^	107 (23.2)	112 (26.6)	330 (53.9)	549 (36.8)
Educational level^b^				
Low	13 (2.8)	18 (4.3)	20 (3.3)	51 (3.4)
Medium	174 (37.7)	159 (37.8)	228 (37.3)	561 (37.6)
High	274 (59.4)	244 (58.0)	364 (59.5)	882 (59.0)

	Mean (SD) (Q1; Q3)	Mean (SD) (Q1; Q3)	Mean (SD) (Q1; Q3)	Mean (SD) (Q1; Q3)
BMI/BMI z-score^c^	0.54 (0.98) (−0.2; 1.2)	0.60 (1.06) (− 0.17; 1.3)	26.6 (5.03) (23.1;29.4)	–
Age (years)	10.7 (1.1) (10.0; 11.1)	14.1 (0.9) (13.2; 15.0)	44.0 (5.1) (40.6; 47.5)	25.3 (16.0) (12.0; 42.5)

^a^Defined by Cole and Lobstein [28]

^b^*ISCED* Highest educational level of the household according to International Standard Classification of Education (ISCED) [22]

^c^BMI for adults and BMI z-scores according to Cole and Lobstein for children and adolescents [28]

### Sweet and fat taste ranking ability and sweet and fat propensity ratings

Across all age groups, most participants had medium sweet or fat taste ranking ability and least participants had high sweet or fat taste ranking ability. Children and adolescents had higher sweet and fat propensity ratios compared to adults (Table 2).

**Table 2:** Distribution of sweet ranking ability, fat ranking ability and sweet and fat propensity ratios (total number (N) and percentages (%) or mean, standard deviation (SD) first and third quartiles (Q25; Q75)) given by age groups

	Children	Adolescents	Adults	Total
	N (%)	N (%)	N (%)	N (%)
Sweet ranking ability				
Low	172 (37.3)	160 (38.0)	220 (36.0)	552 (37.0)
Medium	207 (44.9)	195 (46.3)	292 (47.7)	694 (46.5)
High	82 (17.8)	66 (15.7)	100 (16.3)	248 (16.6)
Fat ranking ability				
Low	155 (33.6)	124 (29.5)	173 (28.3)	452 (30.3)
Medium	206 (44.7)	215 (51.1)	317 (51.8)	738 (49.4)
High	100 (21.7)	82 (19.5)	122 (19.9)	304 (20.4)

	Mean (SD) (Q1; Q3)	Mean (SD) (Q1; Q3)	Mean (SD) (Q1; Q3)	Mean (SD) (Q1; Q3)
Sweet propensity ratio^a^ (%)	21.1 (9.7) (14.2; 26.7)	22.3 (11.2) (14.4; 27.7)	15.8 (10.5) (7.6; 22.7)	19.2 (10.9) (11.6; 25.6)
Fat propensity ratio^b^ (%)	28.24 (9.2) (22.1; 34.0)	25.53 (8.9) (18.9; 31.3)	23.66 (8.7) (17.4; 29.3)	25.60 (9.1) (19.2; 31.6)

^a^Proportion of sweet foods of all consumed foods

^b^Proportion of fatty foods of all consumed foods

### Association of sweet and fat taste ranking ability with sweet and fat propensity ratios

In adults, a negligibly small difference (0.2 percentage points) in sweet propensity ratio between sweet ranking groups was observed (Table 3). The same was true for fat propensity ratio and fat ranking groups (0.4 percentage points) (Table 4).

In adolescents, a difference (1.9 percentage points) in sweet propensity ratios between sweet ranking groups was observed (Table 3) indicating that adolescents with a higher sweet ranking ability had higher sweet propensity ratios. The same was true for fat propensity scores and fat ranking groups (2.2 percentage points) (Table 4).

In children, a difference (1.3 percentage points) in sweet propensity ratios between sweet ranking groups was observed (Table 3) indicating that children with a higher sweet ranking ability had lower sweet propensity ratios. The same was true for fat propensity ratios and fat ranking groups (1.4 percentage points) (Table 4).

**Table 3:** Adjusted^a^ differences in sweet propensity ratios between participants with low sweet ranking ability and medium/high sweet ranking ability (estimates (β) and confidence intervals (CI))

		Sweet propensity ratio (in percentage point)
Children	N	**β **(adjusted mean difference) (CI)
Low sweet ranking ability^b^	172	Reference
Medium or high sweet ranking ability^b^	289	− 1.3 (− 2,9;0.3)
Adolescents	N	β (adjusted mean difference) (CI)
Low sweet ranking ability^b^	160	Reference
Medium or high sweet ranking ability^b^	261	1.9 (− 0.4;4.2)
Adults	N	β (adjusted mean difference) (CI)
Low sweet ranking ability^b^	220	Reference
Medium or high sweet ranking ability^b^	392	− 0.03 (− 1.8;1.8)

*CI* confidence interval

^a^Model is adjusted for sex, age, country, smoking status, ISCED (International Standard Classification of Education [22]) and weight status, random intercept for family affiliation

^b^Exposure is dummy coded (0 for low, 1 for medium or high sweet ranking ability)

**Table 4:** Adjusted^a^ differences in fat propensity ratio between participants with low fat ranking ability and medium/high fat ranking ability (estimates (β) and confidence intervals (CI))

		Fat propensity ratio (in percentage point)
Children	N	β (adjusted mean difference) (CI)
Low fat ranking ability^b^	155	Reference
Medium or high fat ranking ability^b^	306	− 1.4 (− 3.1; 0.3)
Adolescents	N	β (adjusted mean difference) (CI)
Low fat ranking ability^b^	124	Reference
Medium or high fat ranking ability^b^	297	2.2 (0.2; 4.2)
Adults	N	β (adjusted mean difference) (CI)
Low fat ranking ability^b^	173	Reference
Medium or high fat ranking ability^b^	439	0.4 (− 1.1; 2.0)

*CI* confidence interval

^a^Model is adjusted for sex, age, country, smoking status, ISCED (International Standard Classification of Education [22]) and weight status, random intercept for family affiliation

^b^Exposure is dummy coded (0 for low, 1 for medium or high ranking ability)

## Disscussion

### Association of sweet and fat intensity ranking ability with sweet and fat propensity ratios

The present investigation aimed to elucidate whether sweet and fat ranking ability was associated with sweet and fat propensity ratios using data of the I.Family study in children, adolescents and adults from eight different European countries.

We observed no association between sweet and fat ranking ability and reported sweet and fat propensity ratios in adults. Hence, results of our study support earlier findings that fat sensitivity in adults was not associated with fat intake [32]. In contrast, in a study conducted in 69 adult women and another study including 51 adults a low-fat sensitivity was associated with higher fatty food consumption [11, 12]. A possible explanation could be that individuals with a higher fat sensitivity perceive the fatty taste as more intense and individuals that are more sensitive towards a taste sensation need to consume less of it to perceive the same sensation as non-sensitive individuals. The lack of a negative association in adults in our study possibly results from the presence of other factors that influence food propensity ratios in adults. In a previous analysis we revealed that adults with a high educational level consumed less frequently sweet and fat foods [33]. This could imply that educational factors (e.g. nutrition literacy) might have a stronger effect on food choice than intensity perception.

In contrast to adults, children with higher sweet and fat ranking ability had higher sweet and fat propensity ratios. A previous study reported that 11 year old children more sensitive to fat preferred low-fat foods, measured by using a food liking questionnaire [14]. Presuming that a high fat propensity ratio reflect the preference of high fat foods over low fat foods this previous study result aligns to our finding.

Similarly, adolescents who had a higher sweet and fat ranking ability also had higher sweet and fat propensity ratios. This aligns with a previous study in which also a positive association of high sweet sensitivity with higher preference for sweet foods in 38 young adults was found [34]. The authors also stated that this finding was contrary to their hypothesis. Possibly adolescents still prefer more sweet and fatty foods due to the fact that during adolescence external factors become more important. Previous results showed that the dietary intake is more similar among siblings close in age than among parents and their children [35, 36]. Further, peers have an influence on each other’s diets high in sugar and fat [37]. This behaviour seems to be related to the increasing time adolescents spend with digital and social media which in turn exposes them to snack food advertisements [38]. Exposure to food marketing is main driver for higher intakes of sweet and fat foods [39]. Further, it should be noted that adolescents face a phase of insulin resistance during puberty [40]. This could possibly lead to alterations of the sweet taste perception in order to modify sweet tasting food intake.

### Sweet and fat ranking ability of different age groups

Due to the standardised methods we used in our study, we were able to describe sweet and fat ranking ability in a large number of children, adolescents and adults from eight European countries.

Our results show for both taste modalities (sweet and fatty), the highest proportion of participants had medium ranking ability. In another study that involved mothers and children (40 boys and 44 girls between 5 to 10 years) most mothers had high sweet ranking ability, the children were equally distributed across low, medium and high sweet ranking ability. Regarding fat ranking ability, mothers were equally distributed across low, medium and high ranking ability, whereas most children had a low ranking ability [20]. The 84 children included in the mentioned study were between 5 and 10 years old. Our study sample consisted of 461 children aged between 7.9 and 12.0 years and 421 adolescents aged between 12.3 and 17.3 years old. As mentioned before taste perception develops during childhood. Therefore, the differences between our and Mennella et al. (2012) results can be due to the different age-groups. Mennella et al. included only mothers whereas in our study approximately 30% of adults where males. Previous research showed that taste perception between men and women differs [41, 42]. Further, not only taste perception but also the cognitive ability to conduct sensory tests develops during childhood. In a previous study, where we included younger children (6–9 years old) we chose sensory tests demanding less cognitive skills [43].

### Strength and limitations

A particular strength is the measurement of sweet and fat intensity rating across a broad age range in a large pan-European population sample. The strictly standardised data collection across all participating centres adds to this strength. This effort resulted in a large cross-country study sample of families from eight European countries. Furthermore, a broad range of possible confounders was considered in this study. For example, due to collected information about kinship relations we were able to consider family affiliation as random effect in our regression models to adjust for similarities like genetic factors, shared environmental effects and assortative mating [44]. In a future analysis we aim to investigate the heritability of sweet and fat intensity ratings.

Beside those strengths, a few limitations have to be addressed. This analysis was based on cross-sectional data and could not distinguish temporal sequence of taste intensity ratings and food propensity ratios. Parents answered the FFQ for their children under the age of 12 years but they can only report food under parental control. Hence, parents might not have reported everything their children had eaten (e.g. snacks and sweets outside home) and the answers were biased on assumptions about what is socially desired [45]. Additionally, Oliveira et al. revealed that mothers that report the diet of their children tend to report their own intake as their children’s dietary intake [46]. Thus, we acknowledge that multiple reporting errors may be occurring and that these may explain differences observed between the 3 age-groups studied here. Further, the sweet and fat propensity ratios were derived from a qualitative food frequency questionnaire and do not give an indication about portion size or amount eaten but the propensity ratios indicate the tendency to choose sweet and fatty foods over non-sweet and fat foods.

## Conclusion

The present study adds valuable insights into the association between sensory sweet and fat taste intensity ratings and food propensity ratings. It also described for the first time differences in sweet sensitivity and fat intensity ratings sensitivity of children, adolescents and adults across Europe. Our results suggest that the association of taste intensity ratings with food propensity ratios during adolescence differs from the associations we saw in children and adults. This could be due to hormonal changes during puberty, growth and maturation and needs to be investigated in future studies. This observation is important because it suggests the need for age specific strategies and methods to improve healthy diet in children, adolescents and adults.

## Electronic supplementary material

Below is the link to the electronic supplementary material.

Supplementary Material 1 (DOCX 17kb)
