# Supplementary material for: Association of ability to rank sweet and fat taste intensities with sweet and fat food propensity ratios of children, adolescents and adults: the I.Family study
Source: Eur J Nutr. 2024 Dec 11;64(1):42. doi: 10.1007/s00394-024-03538-0 (PMC11634913; doi:10.1007/s00394-024-03538-0)
Supplement: Supplementary file 1 — Supplementary Material 1 (DOCX 17kb) [file 394_2024_3538_MOESM1_ESM.docx]

Supplement table 1: Translations of the labelling of the ß-point-scale to measure sweet and fat intensity ratings from all 8 participating countries

| English | Belgium | Cyprus | Estonia | Germany | Hungary | Italy | Spain | Sweden |
| --- | --- | --- | --- | --- | --- | --- | --- | --- |
| Not at all sweet | Helemaal niet zoet | Καθόλου γλυκό | üldse mitte magus | Überhaupt nicht süß | Egyáltalán nem édes | Per niente dolce | Nada dulce | inte alls sött |
| Very sweet | Zeer zoet | Πολύ γλυκό | väga magus | Sehr süß | Nagyon édes | Molto dolce | Muy dulce | mycket sött |
| Not at all creamy | Helemaal niet romig | Καθόλου κρεμώδες | üldse mitte koorene | Überhaupt nicht cremig | Egyéltalán nem krémes | Per niente cremoso | Nada cremoso | inte alls krämig |
| Very creamy | Zeer romig | Πολύ κρεμώδες | väga koorene | Sehr cremig | Nagyon krémes | Molto cremoso | Muy cremoso | mycket krämig |

Supplement table 2: Results^1^ for the association of sweet and fat ranking ability for three categories with sweet and fat propensity ratios

|  |  | **Sweet propensity ratio** | |
| --- | --- | --- | --- |
| **Children** | **N (%)** | **β (CI)** | |
| Low sweet ranking ability^2^ | 172 (37.3) | Reference | |
| Medium sweet ranking ability^2^ | 207 (44.9) | -1.7 (-3.4;0.1) | |
| High sweet ranking ability^2^ | 82 (17.8) | -0.3 (-2.6;2.0) | |
| **Adolescents** | **N (%)** | **β (CI)** | |
| Low sweet ranking ability^2^ | 160 (38.0) | Reference | |
| Medium sweet ranking ability^2^ | 195 (46.3) | 1.9 (-0.7;4.4) | |
| High sweet ranking ability^2^ | 66 (15.7) | 1.9 (-1.5;5.2) | |
| **Adults** | **N (%)** | **β (CI)** | |
| Low sweet ranking ability^2^ | 220 (36.0) | Reference | |
| Medium sweet ranking ability^2^ | 292 (47.7) | -0.1 (-2.0;1.9) | |
| High sweet ranking ability^2^ | 100 (16.3) | -0.4 (-2.9;2.1) | |
|  |  | | **Fat propensity ratio** |
| **Children** | N (%) | | β (CI) |
| Low fat ranking ability^2^ | 155 (33.6) | | Reference |
| Medium fat ranking ability^2^ | 206 (44.7) | | -1.1 (-3.0;0.7) |
| High fat ranking ability^2^ | 100 (21.7) | | -1.9 (-4.2;0.3) |
| **Adolescents** | N (%) | | β (CI) |
| Low fat ranking ability^2^ | 124 (29.5) | | Reference |
| Medium fat ranking ability^2^ | 215 (51.1) | | 2.9 (0.8;5.0) |
| High fat ranking ability^2^ | 82 (19.5) | | 0.5 (-2.0;3.1) |
| **Adults** | N (%) | | β (CI) |
| Low fat ranking ability^2^ | 173 (28.3) | | Reference |
| Medium fat ranking ability^2^ | 317 (51.8) | | 0.5 (-1.1;2.2) |
| High fat ranking ability^2^ | 122 (19.9) | | -0.0 (-2.0;2.0) |

Abbreviations: CI = Confidence interval
^1^: Model is adjusted for sex, age, country, ISCED (International Standard Classification of Education [15]) and weight status, random intercept for family affiliation
^2^Exposure is dummy coded (0 for low, 1 for medium or high ranking ability)
